# Heterologous Expression of the Cotton NBS-LRR Gene *GbaNA1* Enhances Verticillium Wilt Resistance in *Arabidopsis*

**DOI:** 10.3389/fpls.2018.00119

**Published:** 2018-02-06

**Authors:** Nan-Yang Li, Lei Zhou, Dan-Dan Zhang, Steven J. Klosterman, Ting-Gang Li, Yue-Jing Gui, Zhi-Qiang Kong, Xue-Feng Ma, Dylan P. G. Short, Wen-Qi Zhang, Jun-Jiao Li, Krishna V. Subbarao, Jie-Yin Chen, Xiao-Feng Dai

**Affiliations:** ^1^Laboratory of Cotton Disease, Institute of Food Science and Technology, Chinese Academy of Agricultural Sciences, c/o Key Laboratory of Agro-products Quality and Safety Control in Storage and Transport Process, Ministry of Agriculture, Beijing, China; ^2^Crop Improvement and Protection Research Unit, United States Department of Agriculture, Agricultural Research Service, Salinas, CA, United States; ^3^Department of Plant Pathology, University of California, Davis, Davis, CA, United States

**Keywords:** Verticillium wilt resistance, NBS-LRR, *Arabidopsis thaliana*, R gene, transgenic, ethylene signaling, ROS production

## Abstract

Verticillium wilt caused by *Verticillium dahliae* results in severe losses in cotton, and is economically the most destructive disease of this crop. Improving genetic resistance is the cleanest and least expensive option to manage Verticillium wilt. Previously, we identified the island cotton NBS-LRR-encoding gene *GbaNA1* that confers resistance to the highly virulent *V. dahliae* isolate Vd991. In this study, we expressed cotton *GbaNA1* in the heterologous system of *Arabidopsis thaliana* and investigated the defense response mediated by GbaNA1 following inoculations with *V. dahliae*. Heterologous expression of *GbaNA1* conferred Verticillium wilt resistance in *A. thaliana*. Moreover, overexpression of *GbaNA1* enabled recovery of the resistance phenotype of *A. thaliana* mutants that had lost the function of *GbaNA1* ortholog gene. Investigations of the defense response in *A. thaliana* showed that the reactive oxygen species (ROS) production and the expression of genes associated with the ethylene signaling pathway were enhanced significantly following overexpression of *GbaNA1*. Intriguingly, overexpression of the *GbaNA1* ortholog from *Gossypium hirsutum* (*GhNA1*) in *A. thaliana* did not induce the defense response of ROS production due to the premature termination of *GhNA1*, which lacks the encoded NB-ARC and LRR motifs. *GbaNA1* therefore confers Verticillium wilt resistance in *A. thaliana* by the activation of ROS production and ethylene signaling. These results demonstrate the functional conservation of the NBS-LRR-encoding *GbaNA1* in a heterologous system, and the mechanism of this resistance, both of which may prove valuable in incorporating *GbaNA1*-mediated resistance into other plant species.

## Introduction

Plant resistance (*R*) genes encode products that play a central role in directly or indirectly recognizing effector proteins from pathogens, or in triggering downstream signaling in the innate immune systems in plants (Jones and Dangl, 2006; Zipfel, 2008). Superfamily of R proteins are primarily delineated by the presence, or lack thereof, of a few structural motifs or domains, such as a nucleotide-binding site (NBS), leucine-rich repeat (LRR), Toll/Interleukin-1 receptor (TIR), coiled-coil (CC), transmembrane (Martin et al., 2003; Joshi and Nayak, 2011). Over one hundred *R* genes have been cloned and characterized from a diversity of plant species, collectively conferring resistance to 122 pathogens (Whitham et al., 1994; Hinsch and Staskawicz, 1996; Anderson et al., 1997; Feuillet et al., 1999, 2003; Shen et al., 2007; Sanseverino et al., 2012; Periyannan et al., 2013; Wang et al., 2015; Zhu et al., 2017). Most of these encode nucleotide binding (NB) and C- terminal leucine-rich repeat (LRR) domains, and hence these types of proteins belong to the so-called NB-LRR protein family (Tameling and Takken, 2008; Collier and Moffett, 2009).

Two subclasses of plant NB-LRRs have been characterized and their names are derived from the domain structure at their N-termini. Those that possess a Toll and human interleukin-1 receptor (TIR) domain are referred to as TIR-NB-ARC-LRR or TNL proteins, while those carrying a predicted coiled-coil (CC) domain are classified as CC-NB-ARC-LRR, or CNL proteins (McHale et al., 2006). The two structural units of ARC1 and ARC2, constitute an ARC subdomain in plant NB-LRRs, and combine with the NB domain to form a NB pocket (Tameling et al., 2002; Albrecht and Takken, 2006; Rairdan and Moffett, 2006). The NB-LRR proteins exist in an auto-inhibited state unless the plant is challenged with a pathogen elicitor. NB-LRRs may recognize effector proteins from the pathogens through direct physical interaction (Deslandes et al., 2003; Catanzariti et al., 2010; Krasileva et al., 2010), or indirectly, by detecting modifications of host target proteins that are induced by the effector (Axtell and Staskawicz, 2003; Mackey et al., 2003; van der Hoorn and Kamoun, 2008). The NB-ARC domain of NB-LRRs functions as a molecular switch wherein the ADP-bound state represents the “off” and the ATP-bound state as the “on” state (Moffett et al., 2002; Takken et al., 2006; Collier and Moffett, 2009; Lukasik and Takken, 2009; Slootweg et al., 2013). The conformational change in the NB-ARC domain coincides with the exchange of bound ADP for ATP leading to a stabilization of the active conformation, and subsequent activation of immune signaling pathways (Collier and Moffett, 2009; Lukasik and Takken, 2009; Eitas and Dangl, 2010).

Cultivated cotton is susceptible to Verticillium wilt, a vascular disease that can result in devastating losses of yield and quality. The leaves on infected plants turn yellow or defoliate, and eventually die following infection by *Verticillium dahliae*. In some years, more than 50% of the cotton acreage is affected by Verticillium wilt, significantly reducing the fiber quality and yield (National Cotton Council of America-Disease Database). Efforts to understand the molecular mechanisms of Verticillium wilt caused by *V. dahliae* have been made, including characterization of several genes that contribute to defense responses such as *GbTLP1* (Munis et al., 2010), *GbCAD1* and *GbSSI2* (Gao et al., 2013), *GbRLK* (Zhao et al., 2013), *GbSTK* (Zhang et al., 2013a), *GhPAO* (Mo et al., 2015), *GbSBT1* (Duan et al., 2016), *GbNRX1* (Li et al., 2016), *GbRVd* (Yang et al., 2016), *GaRPL18* (Gong et al., 2017), and GhPGIP1 (Liu N. et al., 2017).

The receptor-like protein encoded by *Ve1* (Verticillium resistance gene 1)-like genes, including *GbVe, GbVe1, Gbvdr5, GbaVd1*, and *GbaVd2*, are homologous to the well-characterized major resistance genes first described in tomato (Zhang et al., 2011, 2012; Yang et al., 2015; Chen et al., 2017). By definition, *Ve* homologs confer resistance to race 1 isolates of *V. dahliae*, which encode the secreted effector Ave1 (de Jonge et al., 2012). The resistance genes activate diverse defense responses following infection by *V. dahliae*, including the regulation of defense hormone (salicylic acid, ethylene, etc.) levels that are involved in spermine and camalexin signaling, enhancing reactive oxygen species scavenging capacity and oxidative stress tolerance, activating the expression of the pathogenesis-related genes, and accelerating phytoalexin (gossypol) synthesis (Gao et al., 2013; Zhang et al., 2013a; Mo et al., 2015; Duan et al., 2016; Yang et al., 2016; Gong et al., 2017; Liu H. et al., 2017). For instance, silencing of *GbNRX1*, a thioredoxin, resulted in defective dissipation of apoplastic ROS, which led to higher ROS accumulation within protoplasts and hence critical for the apoplastic immune response (Li et al., 2016).

Similar to other plant species, the NBS-LRRs comprise a protein superfamily in cotton which encodes at least 300 nucleotide-binding site (NBS) domains and many of these are found encoded in gene clusters in genome (Paterson et al., 2012; Li et al., 2014; Chen et al., 2015). Most (about 76.7%) NBS-encoding genes have undergone striking mutations that reflect an ongoing plant–pathogen “arms race” (Paterson et al., 2012). Comparative genomic analyses showed that tandem duplications may have played a significant role in the expansion of the NBS-encoding gene family in *G. raimondii* (nearly immune to the pathogen) following its divergence from *G. arboretum* (highly susceptible to the pathogen). Correlation analysis revealed that the resistance genes cluster around known Verticillium wilt resistance QTLs, and several of these contain NBS-LRR domains (Chen et al., 2015). However, few NBS-LRR proteins have been reported to function as Verticillium wilt resistance in cotton, except for *GbRVd* (Yang et al., 2016).

The NBS-LRR class gene *GbaNA1* is in the Verticillium wilt resistance locus VdRL08, and confers resistance to the non-race 1 *Verticillium dahliae* isolate Vd991 (Li N.Y. et al., 2017). The *GbaNA1* homolog in *Gossypium hirsutum* prematurely terminates and is non-functional, and is the underlying reason for the susceptibility of *G. hirsutum* (Chen et al., 2015; Li N.Y. et al., 2017). In this study, we further investigated the Verticillium wilt resistance function of *GbaNA1* by heterologous expression in *Arabidopsis thaliana*. The main objectives of the current study were to: (1) study the role of the functional *GbaNA1* in Verticillium wilt resistance in *A. thaliana*; (2) detect whether *GbaNA1* has the ability to recover the function of *GbaNA1* ortholog mutant in *A. thaliana*; (3) explore the defense responses mediated by *GbaNA1* in *A. thaliana*; and (4) use the transgenic *Arabidopsis* to confirm the loss of resistance gene function owing to the truncation of *GbaNA1* homolog in *G. hirsutum*.

## Materials and Methods

### Culture Condition and Inoculation Method

*Arabidopsis thaliana* seedlings were grown in pots with potting soil (PINDSTRUP, Denmark) including 20% vermiculite in a greenhouse at temperatures of 24°C during the day and 20°C at night, 60–70% relative humidity, and under a 16/8 light-dark photoperiod. The highly virulent *V. dahliae* strain Vd991 (used in all experiments) was cultured in potato dextrose broth (PDB) medium at 25°C for 7 days on a shaker. Conidia were harvested by centrifugation and washed with sterile water; the final concentration was adjusted to 5 × 10^6^ conidia/mL using a hemocytometer. For inoculations with *V. dahliae, A. thaliana* seedlings were uprooted, and the roots were dipped in *V. dahliae* conidial suspension for 2 min followed by replanting into vermiculite soil. Verticillium wilt symptoms were recorded 3 weeks after inoculation.

For fungal biomass quantification, stems of three inoculated plants per gene target (one per replicate) were harvested at 21 days post-inoculation. qPCR was performed using a SYBR premix Ex Taq II kit (TaKaRa, Japan) with primers specific to the *A. thaliana* ubiquitin extension protein 1 (*UBQ1*, NM_115119.4) and *V. dahliae* elongation factor 1-α (*EF-1α*) (Supplementary Table S1).

### Gene Cloning

To clone *GbaNA1* (MF078620), 3-week-old seedlings of *Gossypium barbadense* cv. Hai7124 were inoculated with 5 mL of 5 × 10^6^ conidia/mL conidial suspension, and root samples were collected 72 h after inoculation. Total RNA was extracted using a Plant RNA Purification Kit (Tiangen, Beijing, China), and cDNA was synthesized by using a RevertAid^TM^ First Strand cDNA Synthesis Kit from MBI (Fermentas, Glen Burnie, Maryland, MA, United States). Primers were designed according to the full open reading frame (ORF) of the gene Gorai.007323100.1 in the *G. raimondii* reference genome (Paterson et al., 2012; Supplementary Table S1). Primers were used to amplify the target fragment from genomic DNA and cDNA. The PCR conditions consisted of an initial 94°C denaturation step for 10 min, followed by 36 cycles of 94°C for 30 s, 55°C for 30 s, and 72°C for 3 min. PCR products were cloned into the pGEM-T-Easy vector (Promega, Madison, WI, United States) and confirmed by sequencing. *GhNA1* (MF078621) from *G. hirsutum* was sequenced using the same method.

### Sequence Analysis

The ORFs of *GbaNA1* were determined using ORF Finder^1^, and the protein sequences were deduced on the basis of codon sequences. The conserved domains of GbaNA1 were predicted using the InterProScan database (Version 5.21) as described (Li N.Y. et al., 2017). The protein coding region of the NBS-LRR gene (*AT4G27220.1*), orthologous to *GbaNA1* in *G. barbadense*, was acquired by BLASTp analysis using GbaNA1 as a query against *A. thaliana* proteins. The typical motifs of known NB-ARC and LRR domains in AT4G27220.1, including P-loop, RNBS-A, Kinase 2, RNBS-B, RNBS-C, GLPL, RNBS-D and MHD, and that the LRR domain contained 12 imperfect LRRs, were determined by protein sequence alignment with the NB-LRR protein GbaNA1. ClustalX 1.83 software was used for the multiple sequence alignment (Thompson et al., 1997).

### Generation and Analysis of Transgenic *A. thaliana*

The ORF fragments from *GbaNA1* and *GhNA1* (*GbaNA1* allelic gene in *G. hirsutum*) were amplified with primers containing *Sac* I and *BstB* I enzyme sites and were integrated into the binary vector pFAST-G02 under the control of the CaMV35S promoter. The recombinant plasmid (pFAST-G02::*GbaNA1*) was transformed into *Agrobacterium tumefaciens* (strain LBA4404) and introduced into 4-week-old *A. thaliana* (ecotype Col-0) plants using an agrobacterium-mediated floral dip method (Clough and Bent, 1998). Transgenic plants were selected on MS medium containing 50 mg/L Basta, and the T_3_ homozygous transgenic plants were identified with PCR and RT-PCR using the genomic DNA and cDNA samples, respectively; wild-type gDNA and cDNA were used as controls. The amplification conditions consisted of an initial 94°C denaturation step for 10 min, which was followed by 35 cycles of 94°C for 30 s, 55°C for 30 s, and 72°C for 30 s; and *UBQ1* was used as a control. *GbaNA1* was also introduced into the *A. thaliana* mutant At4g27270 (*na1*, SALK_ 127692, the *GbaNA1* ortholog in *A. thaliana*) as described above. The phenotypes of transgenic plants resistant to *V. dahliae* Vd991 were assayed using a root-dip method as described above with 5 mL of *V. dahliae* Vd991 conidial suspension (2 × 10^6^ conidia/mL). The development of fungal biomass in plant tissue was determined by absolute quantification using a method similar to that previously described (Santhanam et al., 2013). In this study, qPCR was performed using SYBR premix Ex Taq II kit (TaKaRa, Japan) with primers specific to the *A. thaliana UBQ1* gene and *V. dahliae* elongation factor 1-α (*EF-1α*).

### ROS Accumulation Detection with DAB Staining

ROS accumulation was detected in transgenic *A. thaliana* and wide-type (Col-0) leaves from 3-week-old plants 12 h after infiltration with 50 μL *V. dahliae* (strain Vd991) conidia suspension (2 × 10^6^ conidia/mL) using 3′3-diaminobenzidine (DAB) solution as previously described (Bindschedler et al., 2006). A sterile water treatment was used as the control. Briefly, the leaves were treated with 1 mg/ml DAB containing 0.05% v/v Tween 20 and 10 mM sodium phosphate buffer (pH 7.0). Then the leaves were incubated at 25°C in the dark, and infiltrated under gentle vacuum. The reaction was terminated at 10–12 h post-inoculation and the DAB solution was removed with a distilled water rinse. Ethanol (75%) was then added to the leaves to remove the chlorophyll and placed in 30% glycerol after the decolorization. Six leaves per treatment were included in each of the three replicates. Samples were observed using a SMZ18 stereo microscope (Nikon, Japan) and the percent of brown pixels of every image from the six leaves examined for each treatment and replication of the same size and resolution was included in obtaining the statistics using the Matlab software.

### Relative Gene Expression Analysis

For the expression analysis of *GhNA1* in cotton, 3-week-old seedlings of *G. hirsutum* Junmian No.1 were inoculated with 5 mL of conidial suspension (5 × 10^6^ conidia/mL) of *V. dahliae* Vd991 using a root-dip method. The inoculated root samples were collected at six time points (2, 6, 12, 24, 48, and 72 h) after inoculation, with three seedlings for each sample. For the expression analysis of ethylene signaling-associated genes and defense response genes in transgenic *A. thaliana*, wild-type (Col-0), *GbaNA1*-overexpression transgenic line (OE1), *GbaNA1* ortholog gene mutant (*na1*), and the transgenic line of *na1* mutant with complemented *GbaNA1* were inoculated with 5 × 10^6^ conidia/mL of *V. dahliae* (strain Vd991) conidia suspension using a root-dip method. Three root samples from each treatment were collected at 24 h after inoculation. RT-qPCR analyses were performed using the SYBR Premix Ex Taq kit (Takara) and a QuantStudio 6 Flex Real Time PCR System (Applied Biosystems, Foster City, CA, United States). PCR conditions consisted of an initial denaturation step at 95°C for 10 min, followed by 40 cycles of denaturation at 95°C for 15 s, annealing at 60°C for 30 s, and extension at 72°C for 30 s. The *A. thaliana UBQ1* was used as endogenous control. All detections were carried out with three independent biological replicates. The relative expression levels of genes were evaluated using the 2^-ΔΔC_T_^ method (Livak and Schmittgen, 2001).

## Results

### Heterologous Overexpression of *GbaNA1* Enhanced Verticillium Wilt Resistance in *A. thaliana*

**FIGURE 1:**
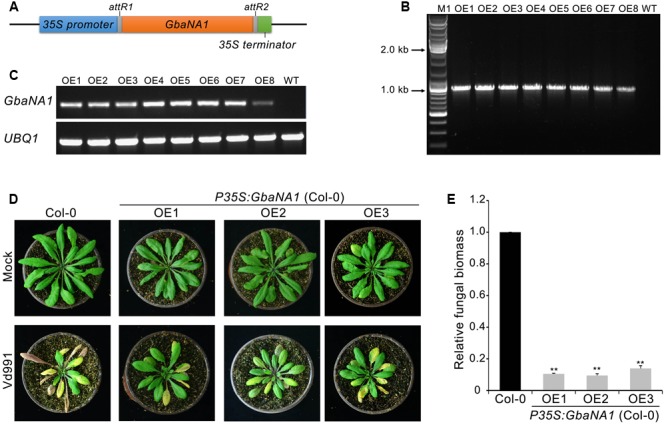
Transgenic expression of *GbaNA1* enhances Verticillium wilt resistance in *Arabidopsis thaliana*. **(A)** Diagram of the *GbaNA1*-overexpressing transformation vector pFAST:GbaNA1. **(B)** PCR products targeting a fragment of *GbaNA1* amplified from DNA extracted from *GbaNA1*-overexpressing transgenic lines of *A. thaliana* ecotype Col-0. **(C)** RT-PCR amplification of *GbaNA1* cDNA in the same transgenic *A. thaliana*. *UBQ1* is shown as a control. **(D)** Phenotype assay of *GbaNA1* transgenic *A. thaliana* inoculated with *V. dahliae*, strain Vd991. *A. thaliana* plants were engineered to express cotton CaMV 35S-driven *GbaNA1* (*P35S:GbaNA1*). Three-week-old seedling of wild-type (Col-0) and transgenic lines (OE1, OE2, and OE3) inoculated with *V. dahliae* strain Vd991 via root-dipping in a suspension of 5 × 10^6^ conidia/mL or sterile water (Mock). Disease symptoms were observed 21 days after inoculation. **(E)** Quantification of *V. dahliae* biomass in *GbaNA1* transgenic *A. thaliana* plants (OE1, OE2, and OE3) compared to the wild-type (Col-0). Relative fungal biomass was determined using quantitative real-time PCR and genomic DNA extracted from total plants of three plants 21 days after inoculation. *V. dahliae* elongation factor 1-α (*EF-1α*) was used to quantify fungal colonization, and *A. thaliana UBQ1* was used as endogenous plant control. Error bars represent standard errors of three biological replicates, ^∗∗^indicates statistical significance (*P* < 0.01), according to unpaired Student’s *t*-tests.

In our previous study, silencing of island cotton NBS-LRR gene *GbaNA1* impaired resistance to the non-race 1 *V. dahliae* isolate Vd991 in cotton (Li N.Y. et al., 2017). To investigate the role of *GbaNA1* in the defense against *V. dahliae*, the gene was heterologously transferred into the *A. thaliana* genome using an *Agrobacterium tumefaciens*-mediated transformation method (Clough and Bent, 1998). The overexpression transformation construct *GbaNA1* driven by the CaMV35S (*35S*) promoter (*P35S:GbaNA1*) was transferred into *A. thaliana* (ecotype *Col-0*) via *Agrobacterium tumefaciens*-mediated transformation (**Figure 1A**). Of the independent T_3_ transgenic lines obtained, the introduced gene could be detected in all eight *GbaNA1*-overexpression lines (OE1 – OE8) using PCR primers specific to *GbaNA1* (**Figure 1B**). Reverse transcription-PCR (RT-PCR) analysis further confirmed that the integrated genes were successfully expressed, as the *GbaNA1* transcript could be detected in the transgenic lines but not in the wild type Col-0 (**Figure 1C**). For Verticillium wilt resistance tests, 4-week-old seedlings of three transgenic lines were arbitrarily selected for inoculation with a highly virulent *V. dahliae* strain Vd991. Relative to the wild-type Col-0, *GbaNA1*-overexpressing lines exhibited significantly enhanced resistance to *V. dahliae* Vd991, as indicated by a reduction of leaf chlorosis and withering (**Figure 1D**). Furthermore, quantitative PCR (qPCR) analysis of fungal biomass demonstrated significantly less *V. dahliae* biomass (<20%) in transgenic plants than in the wild-type plants (**Figure 1E**). These results suggested that the island cotton NBS-LRR gene *GbaNA1* conferred resistance to strain Vd991 after interfamily transfer into *A. thaliana* ecotype Col-0.

### The Orthologous *GbaNA1* Mutant in *A. thaliana* Is Susceptible to *V. dahliae*

**FIGURE 2:**
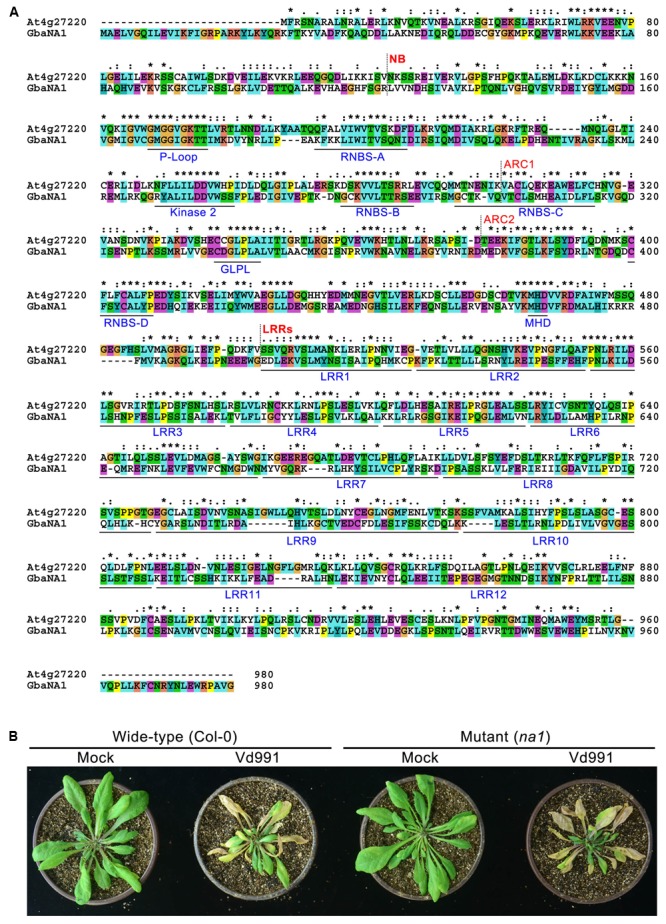
Characteristics of GbaNA1 orthologous proteins in *Arabidopsis thaliana*. **(A)** Protein sequence alignment of GbaNA1 and its orthologous sequence in *A. thaliana* Col-0. The alignment was performed by Clustal X2 with a GONNET 80 protein weight matrix. Asterisks represent conserved resides. **(B)** Verticillium wilt phenotype *A. thaliana* line *At4g27220*, a T-DNA mutant of the gene orthologous to *GbaNA1* (*NA1*). Two-week-old seedlings of *At4g27220* (genotype *na1*) and the wild-type (Col-0) were inoculated with *V. dahliae* strain Vd991 by root-dipping in a suspension of 5 × 10^6^ conidia/mL. Roots were dipped in sterile water as controls (Mock). Phenotypes were investigated 14 days after inoculation.

The *A. thaliana* NBS-LRR gene (AT4G27220.1) is the ortholog of *GraNA1* (Gorai.007G323100.1) in *G. raimondii* (Paterson et al., 2012), and *GraNA1* and *GbaNA1* are allelic between *G. raimondii* and *G. barbadense* (Li N.Y. et al., 2017). BLASTp analysis using GbaNA1 as a query against *A. thaliana* proteins returned AT4G27220.1 as the best hit (Identities = 238/785, 30%; Positives = 389/785, 49%), suggesting that *GbaNA1, G. raimondii* Gorai.007323100.1 and *AT4G27220.1* are orthologous genes. AT4G27220.1 also belongs to the NB-ARC domain-containing disease resistance protein family. Protein sequence alignment showed that although the sequences display significant differences because cotton and *A. thaliana* are phylogenetically divergent, many residues (especially the residues associated with the NB-ARC and LRR domains) were conserved between GbaNA1 and AT4G27220.1 (**Figure 2A**), and contained the typical motifs of known NB-ARC and LRR domain-containing proteins, including P-loop, RNBS-A, Kinase 2, RNBS-B, RNBS-C, GLPL, RNBS-D and MHD, and the LRR domain containing 12 imperfect LRRs (**Figure 2A**).

**FIGURE 3:**
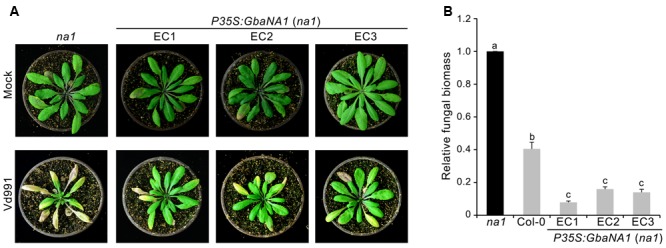
Overexpression of cotton *GbaNA1* in *Arabidopsis thaliana* mutant *At4g27220* (*na1*) enhances the Verticillium wilt resistance of *A. thaliana*. **(A)** Enhancement of the Verticillium wilt resistance of *na1 A. thaliana* mutants by *GbaNA1* overexpression. Overexpression of *GbaNA1* was driven by the CaMV 35S promoter. Three-week-old transgenic lines (EC1, EC2 and EC3) were subjected to a root-dip inoculation in a suspension of 5 × 10^6^ conidia/mL of *V. dahliae*, strain Vd991. Verticillium wilt symptoms were assessed 21 d after inoculation. Sterile water root dips were used as controls (Mock). **(B)** Quantification of *V. dahliae* biomass in transgenic *GbaNA1* overexpression lines of *na1* mutants, *na1* mutant and the wild-type (Col-0). Relative fungal biomass was determined using quantitative real-time PCR and genomic DNA extracted from total plants 21 days after inoculation. *V. dahliae* elongation factor 1-α (*EF-1α*) was used to quantify fungal colonization, and *A. thaliana UBQ1* was used as an endogenous plant control. Error bars represent standard errors of three biological replicates. Different letters above the bars indicate significant differences (*P* < 0.01).

To further confirm whether the *GbaNA1* ortholog gene, *AT4G27220.1* is involved in Verticillium wilt resistance, the response of mutant *AT4G27220.1* (Germplasm/Stock in TAIR: SALK_127692, hereinafter named *na1*) to *V. dahliae* isolate Vd991 was tested using the root dip inoculation method. The pathogenicity assay showed that the mutant line *na1* displayed greater sensitivity to *V. dahliae* compared with the wild-type *Col-0* ecotype, indicated by a significant increase in leaf chlorosis and wilting 2 weeks after inoculation (**Figure 2B**). Investigation of the fungal biomass by qPCR analysis suggested rapid multiplication in the *na1* lines compared to wild-type *Col-0* ecotype (**Figure 3B**). Together, these results showed that the *GbaNA1* ortholog gene *AT4G27220.1* conferred Verticillium wilt resistance in *A. thaliana*.

### Overexpression of *GbaNA1* Reduced Symptom Severity in the AT4G27220 Mutant

To further investigate the involvement of *GbaNA1* in Verticillium wilt defense responses, the sensitivity to *V. dahliae* were assessed in the mutant *na1* of *A. thaliana* after receiving the gene *GbaNA1* driven by the *35S* promoter. The ectopic transformants were verified by PCR and the expression of *GbaNA1* was detected by RT-PCR, and several independent ectopic transformants were obtained, of which three were used for further analysis in this study (Supplementary Figures S1A,B). Inoculation of three separate transgenic *na1* lines complemented with genes *GbaNA1* displayed significantly less chlorosis and wilting compared with the *na1* mutant (**Figure 3A**). Quantification of fungal biomass by qPCR demonstrated that *na1* complemented with *GbaNA1* developed significantly less fungal biomass than the non-complemented *na1* mutants (**Figure 3B**), consistent with constitutive over-expression of *GbaNA1* driven by the *35S* promoter in the wild-type *Col-0* ecotype (**Figure 1D**). These results indicated that *GbaNA1* can restore the Verticillium wilt resistance mediated by the ortholog *AT4G27220.1* in *A. thaliana*, and further confirms the significant role of GbaNA1 in reducing *V. dahliae* colonization and symptom severity.

### Overexpression of *GbaNA1* Enhanced the Defense Response of ROS Activation

**FIGURE 4:**
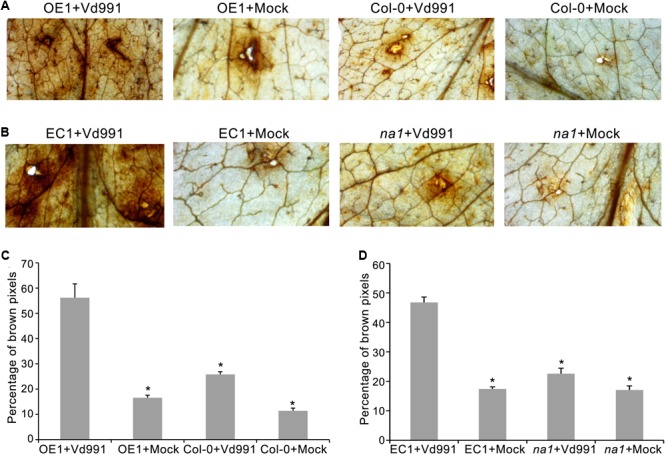
Cotton GbaNA1 modulates ROS accumulation in *Arabidopsis thaliana*. **(A)** The ROS-inducing activities of *GbaNA1* transgenic *A. thaliana* and wide type (Col-0) plants inoculated with *V. dahliae*, strain Vd991. ROS accumulation was assessed in *GbaNA1* transgenic *A. thaliana* and wide type (Col-0) leaves from 3-week-old plants 12 h after infiltration with a 50 μL suspension (5 × 106 conidia/mL) of *V. dahliae*, strain Vd991. Sterile water treatments were used as controls (Mock). **(B)** Detection of ROS-inducing activities of *A. thaliana na1* mutants and *na1* mutants after introduction of *GbaNA1*. **(C)** The percent of brown pixels of *GbaNA1* transgenic *A. thaliana* (OE1) and wild-type (Col-0) plants inoculated with *V. dahliae* strain Vd991. ROS accumulation level was assessed in *GbaNA1* transgenic *A. thaliana* and wide type (Col-0) leaves from 3-week-old plants 12 h after infiltration with a 50 μL conidial suspension (5 × 10^6^ conidia/mL) of *V. dahliae*, strain Vd991 followed by staining with DAB. Sterile water treatments were used as controls (Mock). **(D)** Detection of ROS-inducing level of *A. thaliana na1* mutants and *na1* mutants after the introduction of *GbaNA1*. EC1, the transgenic lines of overexpression *GbaNA1* in *na1* mutant.

To explore the *V. dahliae* defense responses mediated by GbaNA1, ROS accumulation was assessed in leaves of *A. thaliana* ecotype Col-0, *GbaNA1* transgenic Col-0 mutants, *na1* mutants, and *na1* mutants complemented with *GbaNA1* after leaf infiltration with a conidia suspension of *V. dahliae* strain Vd991. Leaves of the wild-type Col-0 displayed more ROS accumulation around infiltration sites (indicated by dark brown deposits visible in leaves) 12 h post-infiltration with conidial suspension, compared to infiltration with sterile water (**Figures 4A,C**). Col-0 *GbaNA1* transgenic plants displayed significantly more ROS accumulation relative to the wild-type Col-0 plants (**Figures 4A,C**). Similarly, overexpression of *GbaNA1* in *na1* mutant plants resulted in higher ROS accumulation than the levels observed in the *na1* mutant plants following inoculation (**Figures 4B,D**). These results demonstrated that *GbaNA1* has the ability to enhance the defense response to *V. dahliae* through *GbaNA1-*mediated ROS activation.

### Ethylene Signaling Is Critical for *GbaNA1*-Mediated Resistance against *V. dahliae*

**FIGURE 5:**
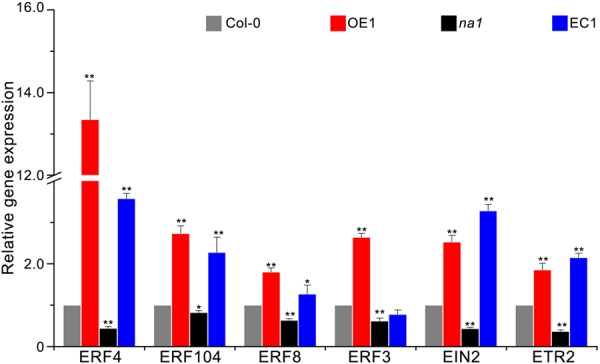
GbaNA1 regulates the expression levels of ethylene signaling-associated genes. Relative expression of six ethylenesignaling-associated genes in *A. thaliana* lines. Wild type Col-0, *GbaNA1*-overexpression transgenic line OE1, *GbaNA1* ortholog mutant *na1*, and a *GbaNA1* overexpression transgenic line of *na1* were inoculated with a suspension of 5 × 10^6^ conidia/mL of *V. dahliae* (strain Vd991) using a root-dip method. Root samples were collected for RNA isolation and cDNA synthesis 24 h after inoculation. Relative expression of ethylene signaling-associated genes was assessed by reverse transcription-quantitative PCR using the comparative threshold 2^-ΔΔCT^ method and *A. thaliana UBQ1* as a reference. Values represent averages of three independent biological replicates. Error bars represent standard errors. Asterisks (^∗^) and double asterisks (^∗∗^) represents statistical significance of *P* < 0.05 and *P* < 0.01, respectively, according to an unpaired Student’s *t*-tests.

Previous results of the expression pattern of *GbaNA1* after treatment with *V. dahliae* strain Vd991 and ET were similar in cotton (Li N.Y. et al., 2017), suggesting that defense responses mediated by *GbaNA1* were also associated with ethylene signaling in *A. thaliana*. To test this association, the relative expression of seven genes (*ERF3, ERF4, ERF13, ERF104, EIN2, ETR2*) in the ethylene signaling pathway was measured using reverse transcription-quantitative PCR (RT-qPCR) in Col-0, *GbaNA1* transgenic Col-0 mutants, *na1* mutants, and *na1* mutants complemented with *GbaNA1* 24 h post-inoculation with *V. dahliae* strain Vd991. Expression levels of six genes (except for *ERF13*) in *GbaNA1* transgenic Col-0 mutants were significantly up-regulated relative to the wild-type plants after inoculation with *V. dahliae* strain Vd991 (**Figure 5**). As expected, the expression of six genes suppressed in *na1* mutant plants (lacking of *AT4G27220.1*) were restored to the relatively high expression level after complementation of the ortholog of *GbaNA1* (**Figure 5**). The relative expression *ERF13* in the ethylene signaling pathway could still be significantly activated by *GbaNA1* after inoculation with *V. dahliae* strain Vd991, but was not affected by the ortholog *AT4G27220.1* in *A. thaliana* (Supplementary Figure S2). Furthermore, the defense response genes *SCL14* and *PR5* were significantly up-regulated after overexpression of *GbaNA1* in the wild-type Col-0 and *na1* mutant plants, but were suppressed in the *na1* mutant plants due to the lack of *GbaNA1* ortholog in *A. thaliana*. These results suggested that ethylene signaling is crucial for *GbaNA1*-mediated defense responses against *V. dahliae* strain Vd991.

### Overexpression of *GbaNA1* Homolog in *G. hirsutum* Results in Loss of Resistance Gene Function

**FIGURE 6:**
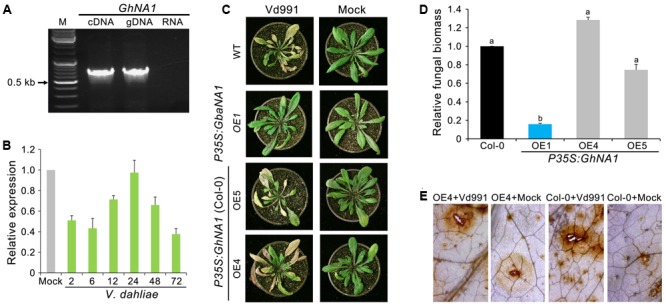
The *GbaNA1* homolog from *G. hirsutum* does not enhance Verticillium resistance in *Arabidopsis thaliana*. **(A)** Cloning *GhNA1* from the Verticillium wilt susceptible *G. hirsutum* cv. Junmian No. 1. *GhNA1* was cloned by reverse transcription-PCR using the cDNA template and genomic DNA, respectively. DNA contamination in the RNA sample was assayed by PCR (RNA lane). **(B)** Expression analysis of *GhaNA1* in *G. hirsutum* cv. Junmian No. 1 after inoculation with *V. dahliae* Vd991. Three-week-old cotton seedlings were root-dipped (2 × 10^6^ conidia/mL) collected over a time-course and RNA was extracted from roots. The relative expression levels of *GhNA1* were assessed by quantitative reverse transcriptase PCR, using the *A. thaliana UBQ1* gene as a reference. Plants treated with sterile water were used as controls (Mock). Error bars represent standard errors of three biological replicates. **(C)** Verticillium wilt symptoms of *GhNA1* transgenic *A. thaliana* inoculated with *V. dahliae*. Two independent transgenic lines of 3-week-old Col-0 seedlings were inoculated with *V. dahliae* (strain Vd991) via root-dipping in a suspension of 5 × 10^6^ conidia/mL. The *GbaNA1* transgenic line (OE1) and wide type (Col-0) served as positive and negative controls, respectively. Mock inoculations were performed with sterile water. **(D)** Quantification of *V. dahliae* biomass in *GhNA1* transgenic *A. thaliana* plants (OE4, OE5, and OE6) compared to the wild type (Col 0). Fungal biomass was determined by quantitative real-time PCR using genomic DNA extracted from whole plants 21 days after inoculation. Error bars represent standard errors of three biological replicates. Columns with different letters indicate statistical significance (*P* < 0.01), according to unpaired Student’s *t*-tests. **(E)** Detecting the ROS-inducing activities of *GhNA1* transgenic *A. thaliana*. Leaves from 3-week-old plants were visualized 12 h after infiltration with 50 μL *V. dahliae*, strain Vd991 (5 × 10^6^ conidia/mL). Sterile water treatment served as a control (Mock). Leaves were stained with DAB.

A previous study revealed high allelic divergence in *GbaNA1*, between the homologs of the Verticillium wilt susceptible *G. hirsutum* and the resistant *G. barbadense* (Li N.Y. et al., 2017). The premature termination of the protein encoded by *GbaNA1* homologs in the *G. hirsutum* accessions results in a lack of the most conserved motifs in the NB-ARC domain (Li N.Y. et al., 2017). Cloning the *GbaNA1* homolog (*GhNA1*) from *G. hirsutum* accessions following RT-PCR confirmed that the coding sequence consisted of 756 bp (**Figure 6A**) which would encode a product of 251 aa residues in length. RT-qPCR analysis showed that *GhNA1* was not responsive to infection with *V. dahliae* strain Vd991 (**Figure 6B**). To assess the relationship between transgenic *GhNA1* plants and Verticillium wilt resistance, *GhNA1*-overexpressing *A. thaliana* transgenic lines were generated, and the positive ectopic transformants, and those expressing the gene, were determined by PCR and RT-PCR (Supplementary Figures S1C,D), respectively. Unlike the *GbaNA1*-overexpressing lines, which displayed enhanced the resistance to *V. dahliae*, the *GhNA1*-overexpressing lines displayed no enhanced resistance against *V. dahliae* strain Vd991, and the disease symptom severity and *in planta* fungal biomass were not significantly different from the wild-type Col-0 inoculated with *V. dahliae* (**Figures 6C,D**). In addition, the accumulation of ROS in the *GhNA1* transgenic lines was also similar to the wild-type Col-0 12 h after infiltration with a suspension of *V. dahliae* conidia (**Figure 6E** and Supplementary Figure S3). These results suggested that *GhNA1* of *G. hirsutum* no longer confers resistance to Verticillium wilt due to its premature termination, unlike the allele from *G. barbadense* cultivars, which would yield the full-length protein product.

## Discussion

Improving genetic resistance is the preferred method to manage Verticillium wilt in most crops (Schaible et al., 1951; Putt, 1964; Simko et al., 2004; Bolek et al., 2005; Zebrowska et al., 2006), but is also the most difficult to implement because of the general lack of effective resistance genes against this disease. In our previous study, we identified an island cotton NBS-LRR gene *GbaNA1*, which conferred resistance to the non-race 1 *V. dahliae* strain Vd991, and found that the *GbaNA1* homolog in susceptible *G. hirsutum* displayed premature termination and was therefore non-functional (Li N.Y. et al., 2017). In this study, we investigated the Verticillium wilt resistance function of *GbaNA1* by ectopic expression in *A. thaliana*, and found that ROS activation and ethylene signaling were critical for GbaNA1-mediated resistance against *V. dahliae*.

Several NB-LRR genes have been identified to function as *R* genes in *A. thaliana*, including *RPM1, RPS1, RPS2, RPS4*, and *RPS5* (Lee and Yeom, 2015). In cotton, the genome has an expanded repertoire of NBS-encoding genes (Xiang et al., 2017), even notable in the diploid genome of *G. raimondii* that encodes more than 300 of these types of genes (Paterson et al., 2012). Comparative genomic analysis showed that the NBS-encoding genes are significantly expanded in *G. raimondii*, which is nearly immune to Verticillium wilt, as compared to *G. arboretum*, which is susceptible to Verticillium wilt (Li et al., 2014). Large scale transcriptome analysis of a cotton response to *V. dahliae* revealed that the NBS-encoding genes may be involved in Verticillium wilt resistance (Xu et al., 2011, 2014; Sun et al., 2013; Zhang et al., 2013b, 2017; Shao et al., 2015). However, few of the candidate NBS-encoding genes involved in Verticillium wilt resistance have been cloned or studied in cotton, except for the NBS-LRR-encoding *GbRVd* (Yang et al., 2016).

Identification of effective Verticillium wilt resistance genes is difficult because of the complexity of the cotton genome and aggressive pathogenicity of *V. dahliae* on most cultivars, although at least 80 different Verticillium wilt resistance quantitative trait loci (QTLs) have been reported on cotton (Wang et al., 2007, 2008, 2012; Yang et al., 2008; Jiang et al., 2009; Zhao et al., 2014). At present, the homology-based cloning or differential expression screening are generally employed to clone Verticillium wilt resistance genes, and several genes have been identified and proven to play important roles during *V. dahliae* infection on cotton (Munis et al., 2010; Gao et al., 2013; Zhang et al., 2013b; Zhao et al., 2013; Mo et al., 2015; Duan et al., 2016; Li et al., 2016; Yang et al., 2016; Gong et al., 2017; Liu H. et al., 2017). However, only a few of these have the *R* gene characteristics.

Recently, several genes possessing the *R* gene characteristics of the receptor-like proteins were cloned from *G. barbadense* using homology-based cloning methods (Zhang et al., 2011, 2012; Yang et al., 2015; Chen et al., 2017), following the discovery of the tomato receptor like protein *Ve1* that specifically mediates the resistance to the *V. dahliae* race 1 strain (Kawchuk et al., 2001; Fradin et al., 2009). In our previous study, 26 *Verticillium dahliae* resistance loci (VdRLs) were identified in *G. barbadense* by the bioinformatics-driven method based on the resistance gene analogue (RGAs) clusters and their transcriptome (Chen et al., 2015). Finally, we obtained the NBS-LRR gene (*GbaNA1*) from the VdRL08 locus that is involved in Verticillium wilt resistance (Li N.Y. et al., 2017). Compared with the resistant *G. barbadense*, the premature termination of the protein encoded by *GbaNA1* homologs in the Verticillium wilt susceptible *G. hirsutum* accessions resulted in a truncated protein that lacks the most conserved motifs in the NB-ARC domain (Li N.Y. et al., 2017). These conserved motifs are important for NB-LRR function in disease resistance (Tameling et al., 2002; Tornero et al., 2002; Williams et al., 2011; Wang et al., 2015). To our knowledge, *GbaNA1* is the first typical NBS-LRR protein to be involved in Verticillium wilt resistance in cotton, and an ortholog can be functional in another plant family, as confirmed in this study using *A. thaliana* transgenic lines.

The recognition of a specific pathogen effector (elicitor) by a corresponding R protein, including the NBS-LRR protein encoded by *R* genes, can initiate a cascade of defense responses, including a hypersensitive response, ROS production, hormone synthesis and signaling transport, and activation of defense-related genes (De Young and Innes, 2006; Caplan et al., 2008; Elmore et al., 2011). In the case of NBS-LRR proteins, tomato Mi-1.1 and 1.2 have been shown to play dual regulatory roles in regulating host cell death (Lukasik-Shreepaathy et al., 2012). The overexpression of *VaRGA1* in *Nicotiana benthamiana* conferred enhanced resistance to *Phytophthora parasitica* through the activation of salicylic acid (SA) signaling and phenylpropanoid pathways (Li X. et al., 2017). In cotton, defense responses including hormone signaling, ROS scavenging and activation of the pathogenesis-related gene expression, were all proven to play roles in Verticillium wilt resistance (Gao et al., 2013; Zhang et al., 2013a; Mo et al., 2015; Duan et al., 2016; Yang et al., 2016; Gong et al., 2017; Liu H. et al., 2017). The NBS-LRR protein GbaNA1 can be significantly induced following treatment with the ethylene (Li N.Y. et al., 2017), and several genes encoding ethylene-responsive element-binding factor were significantly up-regulated after *GbaNA1* overexpression in *A. thaliana*. Furthermore, ROS production was also significantly increased in *GbaNA1*-overexpressing *A. thaliana* lines compared with the wild-type plants. Ethylene and ROS are important signaling molecules mediating numerous important biological processes (Zhang et al., 2016). In *A. thaliana*, the crosstalk between the ethylene and ROS accumulation leads to stomatal closure and associated immunity after infection with *Pseudomonas syringae* (Mersmann et al., 2010). Activation of ethylene signaling pathways also enhances disease resistance by regulating ROS and phytoalexin production in rice during infection by *Magnaporthe oryzae* (Yang et al., 2017). Defense responses of ethylene signaling activation and increased ROS production can be mediated by the NBS-LRR proteins. For instance, overexpression of rice NBS-LRR resistance gene, *OsBIHD1*, resulted in enhanced expression of the ethylene synthesis genes involved in ethylene-mediated immunity (Liu H. et al., 2017); and the wheat NBS-LRR protein TaRCR1 regulating certain reactive oxygen species (ROS)-scavenging and production, play important roles in plant defense responses to the necrotrophic fungal pathogen, *Rhizoctonia cerealis* (Zhu et al., 2017). The activation of ethylene signaling is also evidenced by the negligible growth of transgenic lines relative to the wild-type plants (**Figure 1D**). Ethylene is a developmental regulator that is involved in manifold physiological processes throughout the plant life cycle (Iqbal et al., 2017). Together, this study of *GbaNA1* in transgenic *A. thaliana* supports the hypothesis that ethylene signaling and increased ROS production are important for *GbaNA1*-mediated resistance against *V. dahliae*.

## Conclusion

Our study found that heterologous overexpression of *GbaNA1* enhanced Verticillium wilt resistance in *A. thaliana*, resulting in the activation of defense responses of ROS accumulation and genes associated with the ethylene signaling pathway. In *A. thaliana*, the NBS-LRR gene *AT4G27220.1* is orthologous to *GbaNA1*, and the *AT4G27220.1* mutant (*na1*) was susceptible to *V. dahliae*. Overexpression of *GbaNA1* in *na1* mutant plants restored activation of the expression of ethylene signaling pathway-related genes and increased ROS production, resulting in the restoration of *A. thaliana* resistance to *V. dahliae*. Moreover, heterologous expression of the *GbaNA1* homolog from *G. hirsutum* (*GhNA1*) in *A. thaliana* could not activate the defense responses and enhance Verticillium wilt resistance, due to the truncation of *GhNA1* and its product that lacks the conserved NB-ARC domain and LRR domain motifs. These results indicate that *GbaNA1* encodes a structural R protein that confers Verticillium wilt resistance by mechanisms that are conserved across some plant families.

## Author Contributions

X-FD, J-YC, and KS conceived and designed the experiments. N-YL, LZ, D-DZ, T-GL, and Y-JG performed the experiments. Z-QK, W-QZ, J-JL, and X-FM prepared biological material. X-FD and J-YC wrote the original draft. SK, DS, and KS edited and re-wrote parts of the manuscript.

## Conflict of Interest Statement

The authors declare that the research was conducted in the absence of any commercial or financial relationships that could be construed as a potential conflict of interest.

## Footnotes

1 https://www.ncbi.nlm.nih.gov/orffinder/
