# Supplementary material for: Heterologous Expression of the Cotton NBS-LRR Gene GbaNA1 Enhances Verticillium Wilt Resistance in Arabidopsis
Source: Front Plant Sci. 2018 Feb 6;9:119. doi: 10.3389/fpls.2018.00119 (PMC5808209; doi:10.3389/fpls.2018.00119)
Supplement: Supplementary file 2 [file Presentation_1.pdf]

Supplementary Figures

Heterologous Expression of the Cotton NBS-LRR Gene *GbaNA1* Enhances Verticillium wilt Resistance in *Arabidopsis*

Authors:

Nan-Yang Li[1,*], Lei Zhou[1,*], Dan-Dan Zhang[1,*], Steven J. Klosterman[3], Ting-Gang Li[1], Yue-Jing Gui[1], Zhi-Qiang Kong[1], Xue-Feng Ma[1], Dylan P. G. Short[2], Wen-Qi Zhang[1], Jun-Jiao Li[1], Krishna V. Subbarao[2,#], Jie-Yin Chen[1,#], Xiao-Feng Dai[1,#]

[*]These authors contributed equally to this work

Institutional affiliation:

[1] Laboratory of Cotton Disease, Institute of Food Science and Technology, Chinese Academy of Agricultural Sciences, Beijing, 100193, China

[2] Department of Plant Pathology, University of California, Davis, c/o U.S. Agricultural Research Station, Salinas, California, United States of America

[3]United States Department of Agriculture, Agricultural Research Service, Crop Improvement and Protection Research Unit, Salinas, CA, USA

The corresponding authors:

[1] Xiao-Feng Dai and Jie-Yin Chen

The Institute of Food Science and Technology

Chinese Academy of Agricultural Sciences

Beijing 100193, P.R. China

Fax: +86-10-62813566;

E-mail: daixiaofeng_caas@126.com, chenjieyin@caas.cn;

[2] Krishna V. Subbarao

E-mail: kvsubbarao@ucdavis.edu

Running title: **The Island Cotton NBS-LRR Gene**

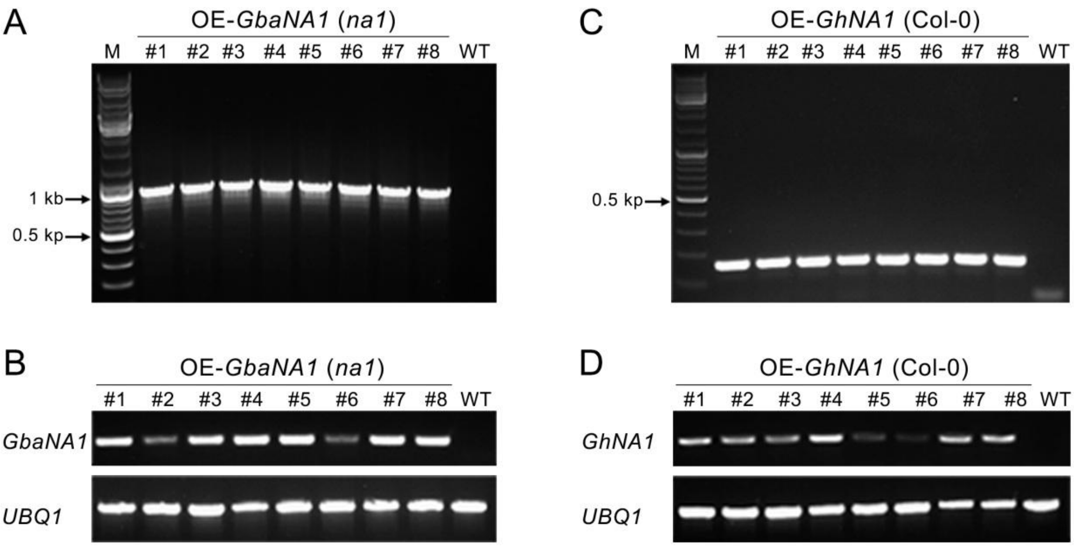

Figure S1 | Identification of the positive *Arabidopsis thaliana* transgenic lines. (**A**) PCR products of *GbaNA1*, amplified from DNA extracted from *GbaNA1*-overexpressing transgenic lines of *A. thaliana na1* mutant. (**B**) Reverse transcription-PCR (RT-PCR) amplification of *GbaNA1* cDNA in the same transgenic *A. thaliana*. *UBQ1* is shown as a control. (**C**) PCR products of *GhNA1*, amplified from DNA extracted from *GhNA1*-overexpressing transgenic lines of *A. thaliana* ecotype Col-0. (**D**) RT-PCR amplification of *GhNA1* from cDNA prepared from the same transgenic *A. thaliana*. *UBQ1* is shown as a control.

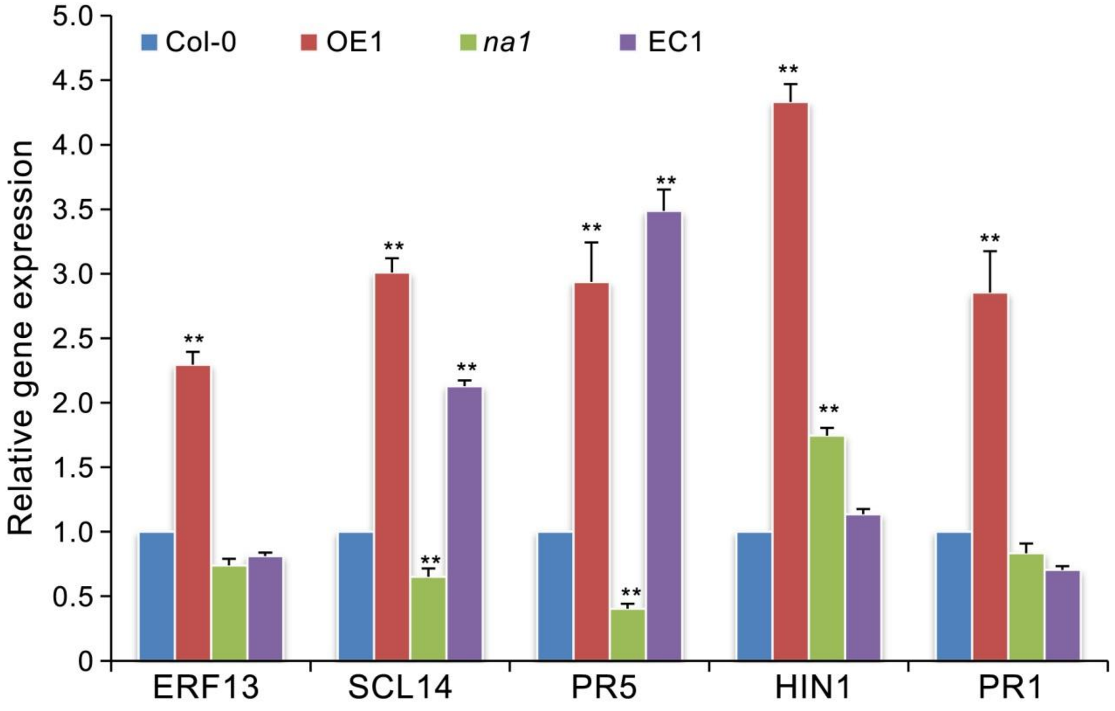

Figure S2 | Detected the expression levels of ethylene signaling-associated gene and defense response genes. Relative expression levels of one ethylene signaling-associated gene (ERF13) and four defense response genes (*SCL14*, *HIN1*, *PR1* and *PR5*) in *A. thaliana* lines. Col-0, *A. thaliana* ecotype Col-0; OE1, *GbaNA1*-overexpression transgenic line; *na1*, *GbaNA1* ortholog mutant; EC1, a *GbaNA1* overexpression transgenic line of *na1*. Relative expression was assessed by reverse transcription-quantitative PCR using the comparative threshold $2^{-\Delta\Delta CT}$ method and *ubiquitin 4* as a reference. Values represent averages of three independent biological replicates. Error bars represent standard errors. Double asterisks (**) represents statistical significance of $P < 0.01$, according to unpaired Student's *t*-tests.

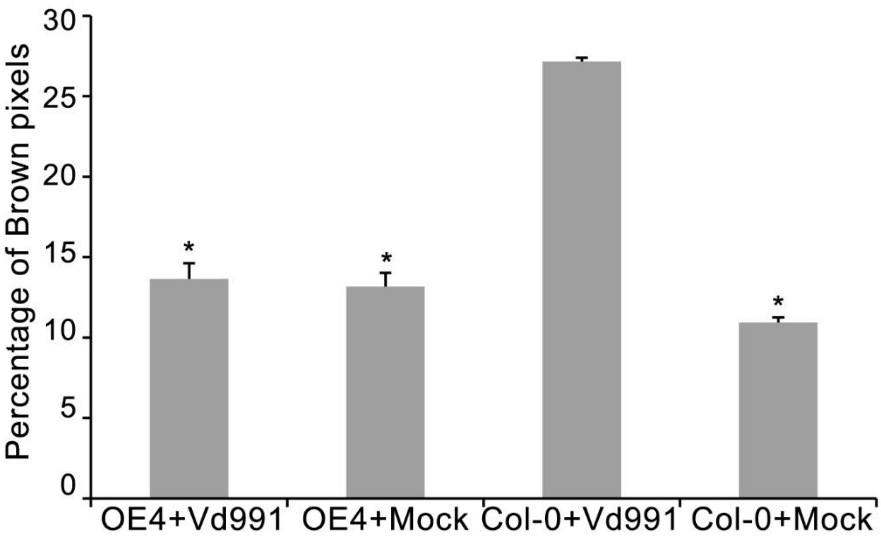

Figure S3 | Detected the ROS accumulation level in *GhNA1* transgenic *Arabidopsis thaliana*. The percent of brown pixels of *GhNA1* transgenic *A. thaliana* (OE4) and wide type (Col-0) plants inoculated with *V. dahliae* strain Vd991. ROS accumulation level was assessed in *GhNA1* transgenic *A. thaliana* and wide type (Col-0) leaves from 3-week-old plants 12 h after infiltration with a 50 μL conidial suspension (5×10^6 conidia/mL) of *V. dahliae*, strain Vd991 followed by staining with DAB. Sterile water treatments were used as controls (Mock).
